# Calibration-free assays on standard real-time PCR devices

**DOI:** 10.1038/srep44854

**Published:** 2017-03-22

**Authors:** Pawel R. Debski, Kamil Gewartowski, Seweryn Bajer, Piotr Garstecki

**Affiliations:** 1 Institute of Physical Chemistry, Polish Academy of Sciences, Kasprzaka 44/52, Warsaw, 01-224, Poland; 2 Curiosity Diagnostics Sp. z o.o., Kasprzaka 44/52, Warsaw, 01-224, Poland

## Abstract

Quantitative Polymerase Chain Reaction (qPCR) is one of central techniques in molecular biology and important tool in medical diagnostics. While being a golden standard qPCR techniques depend on reference measurements and are susceptible to large errors caused by even small changes of reaction efficiency or conditions that are typically not marked by decreased precision. Digital PCR (dPCR) technologies should alleviate the need for calibration by providing absolute quantitation using binary (yes/no) signals from partitions provided that the basic assumption of amplification a single target molecule into a positive signal is met. Still, the access to digital techniques is limited because they require new instruments. We show an analog-digital method that can be executed on standard (real-time) qPCR devices. It benefits from real-time readout, providing calibration-free assessment. The method combines advantages of qPCR and dPCR and bypasses their drawbacks. The protocols provide for small simplified partitioning that can be fitted within standard well plate format. We demonstrate that with the use of synergistic assay design standard qPCR devices are capable of absolute quantitation when normal qPCR protocols fail to provide accurate estimates. We list practical recipes how to design assays for required parameters, and how to analyze signals to estimate concentration.

Here we demonstrate a new type of assay that synergistically combines the advantages of digital and analogue quantitative PCR. The combination of the digital and analogue information provides for absolute quantitation with adjustable resolution. We also describe the procedure and derive a prescription for designing synergistic digital-analogue PCR assays and for analyzing the results. The assays that we describe in this report can be used to analyze difficult and exotic samples on standard qPCR (real-time) devices and to design new digital-analogue assays that maximize information gain from the partitions and thus reduce to minimum their number required to reach a particular level of precision in a given dynamic range of concentrations.

The analogue qPCR (in the format of Real-Time PCR^1^,^2^,^3^,^4^) was introduced in 1991 by Holland *et al*.^5^ (Fig. 1A). The technique traces the increase of intensity of fluorescence from a stained DNA product during PCR. Given that in each cycle the number of copies of the PCR product (the amplicons) increases q-folds, the concentration *C*_*p*_ of the product increases geometrically *C*_*p*_ ∝ *q*^*n*^ in the cycle number n. If the product is fluorescent, one can assess the initial concentration C of the target strain by comparing the number of cycles needed to reach a threshold intensity of light between the sample and calibrated references.

qPCR can be used to determine the concentration of target DNA over a wide range of initial concentrations of the target. The fact that the estimate relies on comparison to a reference compromises the accuracy and precision of the measurement by variation in multiple experimental factors, such as quality of the reference samples, the type and quality of the buffer, the activity of the polymerase, the amount and specificity of primers, and the reproducibility of thermal cycling and/or of detection in the apparatus.

In spite of these limitations, qPCR remains the ‘golden standard’. The advantages include simple liquid handling protocols that do not require extensive partitioning of the sample, and straightforward mathematical procedures for obtaining the final result from the measurements on the sample and from the calibration curve.

## Digital PCR

**Figure 1:**
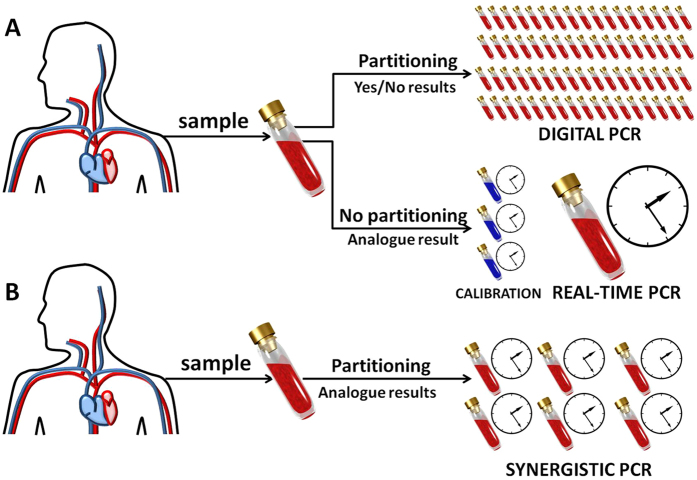
A scheme of the analytical procedures. (**A**) dPCR and qPCR assays. Digital assays require the division of the sample into a large number of partitions, even thousands to millions, but they provide assessment without calibration. Real-Time assays require only one partition but need calibration using standardized samples to give an absolute result. (**B**) Synergistic assays described in this paper require simplified partitioning of the sample – only tens of non-identical partitions are needed – and provide absolute assessment without calibration.

The idea of digital protocols root back to 1915 when McCrady^6^ introduced the limiting-dilution assay. He proposed the mathematical model—the most probable number method—for quantization of bacterial cells. Application of the digital protocols in quantitative assessment based on PCR was first proposed in 1992 by Sykes *et al*.^4^. The design proposed comprised 60 compartments grouped into six sets, each set being three-fold diluted to create a geometric sequence of the number of DNA copies from the sample. This idea was further developed in 1999 by Vogelstein and Kinzler. They proposed using identical partitions yielding either a positive (*s* = 1) or negative (*s* = 0) signal if the partition contained at least one target molecule (*m* ≥ 1) or not (*m* = 0). As the assessment of the concentration by digital assays relies solely on the presence of a signal, and not on its analogue value, the resulting estimate of concentration is absolute i.e. not relying on comparison to any reference experiment (Fig. 1A). dPCR assays are typically also highly precise and sensitive^3^,^7^,^8^,^9^,^10^. The digital schemes require strong amplification of the signal (i.e. number of molecules of the analyte), hence the digital scheme is applicable predominantly to qPCR^7^,^8^,^9^,^10^ and quantitative immuno-assays^11^,^12^.

The digital assays are hoped to replace the standard analogue procedures. They provide absolute quantification without calibration of the experimental set-up. Still, it has to be ensured that no false positive or false negative signals are present due to the contamination of the sample or the limited amplification efficiency and specificity. They may also benefit from simplified execution of the amplification and detection protocols for end-point measurements (which may be simpler compared to real-time tracking of the signal). Yet the spread of the digital techniques in research and in diagnostics is slow due to the cost and complexity of the devices and protocols, as they require partitioning of the sample into large (or sometimes astronomically large) number of test volumes (typically thousands to millions partitions)^13^,^14^,^15^. Furthermore, the precision and the dynamic range of a classic digital assay comprising identical partitions of the sample cannot be tuned independently. It is not, for example, possible to obtain high precision (low standard deviation of the estimate of concentration) in a narrow range of concentrations while using a small number of compartments, or vice-versa, to obtain an imprecise estimate over a wide dynamic range. We have recently shown^16^ a method for optimization of information gain from digital signals and for an optimum (rational) design of assays that minimizes the number of compartments required to obtain a given precision over a requested dynamic range. The protocol offers dramatic reduction (by orders of magnitude in comparison to classic digital methods) of the number of partitions. These end-point digital assays could be run on qPCR devices that use the strips (32) or (96, 192 or 384) well plates.

## The potential for synergy

The existing methods of quantitative assessment based on PCR (dPCR and qPCR) present complementary features that have not yet been combined. The qPCR techniques offer relatively facile handling of samples. The sample is typically mixed with the PCR kit into one well. The increase of the real-time fluorescent signal is then compared with a calibration curve obtained from a reference sample. The method offers high information gain via analogue resolution of the signal yet relies heavily on calibration - an effect that may introduce significant errors in accuracy of the result. The digital assays alleviate the need for calibration, yet due to the intrinsically low (binary) information content of the signal from any single partition, the classic digital assays that use identical compartments require large numbers of them.

While the number of required partitions of the sample in a digital assay can be minimized^7^,^8^,^9^,^10^,^16^ while using only binary (yes/no) signals, here we show further reduction of the number of partitions of the sample or, alternatively, an enhancement of the precision of the assessment, obtained via a synergistic analysis of both the digital and analogue signals recorded on a qPCR instrument. The synergy combines the absolute character stemming from the digital analysis with precision and dynamic range delivered by the analogue (Real-Time) signals. The synergistic assay combines the information from the digital and from the analogue signals to improve the precision of the estimate (Fig. 1B). Below we derive the synergistic assay step by step. Say that the sample contains the target analyte at (unknown) concentration *C*. A digital test, conducted on any isolated portion of the sample, provides information whether the partition, characterized by a volume *v*_*i*_ and a dilution factor *d*_*i*_ (with *C*_*i*_ = *d*_*i*_*C* being the expected concentration of sample in the *i*-th compartment), contained a number *m*_*i*_ of the target molecules equal or greater than a threshold number *m*_*tr*_ (e.g. *m*_*tr*_ = 1). The digital test results in recording either a positive value of the signal that confirms the hypothesis (e.g. *m*_*i*_ ≥ *m*_*tr*_) or a negative value otherwise.

## Information content of a binary signal

**Figure 2:**
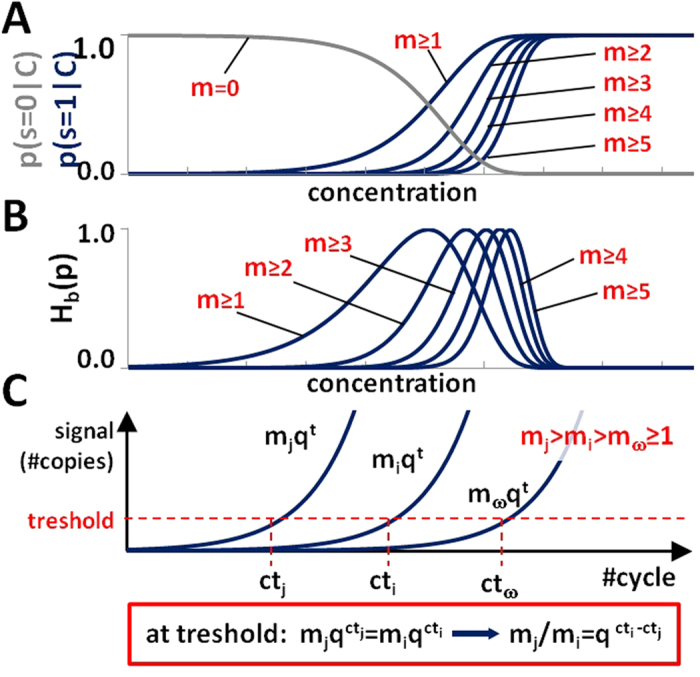
(**A**) Probability 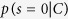 (grey line) of not finding any target molecules in a test-volume (negative digital signal *s* = 0 and probabilities 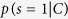 (blue lines) of finding at least a threshold number of molecules in a test-volume (positive digital signal *s* = 1. For dPCR, the threshold number of molecules is typically equal to one (*m* ≥ 1). Using synergistically information from digital and analogue readout, one can interpret positive signals as the threshold number was higher, which effectively shifts the probability functions towards the real value of initial concentration. Bayesian formalism translates the signal into probabilistic information 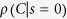 and 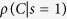 on *C*, later used for the more precise calculation of the estimate of initial concentration. (**B**) The Shannon (binary) Entropy function *H*_b_ (p) quantifies the information gain from a single Bernoulli trial (i.e. a test with that provides only a positive/negative answer). Each trial provides most information at a specific value of concentration 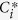, which depends solely on the volume and dilution of a test-volume *d*_*i*_*v*_*i*_, and a threshold number of molecules *m*_*tr*_ (i.e. for *m*_*tr*_ = 1, 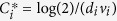. (**C**) Measurement of the real-time signal. In the qPCR, the level of signal (i.e. fluorescence) that is proportional to the number of copies of the amplicon in the compartment is measured at predefined time intervals, preferably at the end of every PCR cycle. The procedure returns an approximation (or estimation) of the real (non-integer) number of the cycle at which the signal from a given compartment crosses the pre-set threshold. Such identified threshold cycle number corresponds to a given, fixed (unknown, but same for all positive compartments) number of copies of the amplicons in that compartment. This allows using simple calculations to determine the ratios of initial numbers of molecules in these compartments as a function of their threshold cycle number *ct*_*i*_.

The fact that the test volume (partition) contained a threshold number of molecules of analyte (or not) conveys a probabilistic information about the initial concentration *C*. For example, under the assumption that the initial number of molecules *m*_*i*_ in the partition is given by a Poissonian distribution 
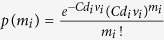
, the positive signal can be translated into a density of probability that a given initial concentration *C* has caused the recording of a positive signal from the partition: 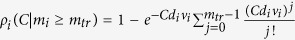. For example, for *m*_*tr*_ = 1, 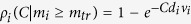. Similarly, a negative signal can be translated into 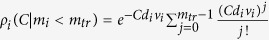.For example, for *m*_*tr*_ = 1, 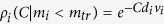. The resulting sigmoidal probability functions are centered (i.e. present the value of 1/2) at a concentration C^*^. This concentration is characteristic of the partition only, i.e. in the example of *m*_*tr*_ = 1, at 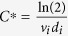 (Fig. 2A) and does not depend on the (stochastic) value of the actual concentration of the analyte in the sample. The measure of the information gained from a single signal (Bernoulli trial) in a digital assay can be extracted with tools derived from the information theory. One such measure is the Shannon entropy 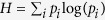, where *i* counts the two (positive/negative) possible outcomes (Fig. 2B). In the dPCR, the efficiency of amplification of the presence of even a single-molecule of the analyte in the given partition is assumed to be 100%. Under this assumption *H* achieves maximum exactly at concentration 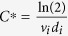. The information gain from the measurement of the state of a test-volume is highest at concentrations near C^*^, for which 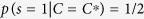 (Fig. 2A), and low for much smaller or much larger concentrations.

## Information content of digital assay

In a purely digital assay (that uses only the end-point positive/negative signals) the estimate of *C* is given by the product of all the probability densities obtained from individual compartments: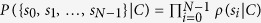. The resulting distribution yields the estimate of the initial concentration of the analyte. This information can be expressed with the Bayesian formalism as the probability *ρ(C*) that a given value of *C* has caused the recorded outcome. The lack of prior knowledge of *C* can be encoded with an initially constant density of probability *ρ*_0_(*C*) = 1/*C*_∞_ of finding any particular value of *C* between *C* = 0 and an arbitrary upper bound *C*_∞_. Then the information about *C* gained from a single signal (*s* = 1 or *s* = 0) is 
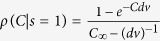
 and *ρ(C*|*s* = 0) = *dve*^−*Cdv*^ (Fig. 2A).

Since the sigmoidal contributions from the compartments *p*_*i*_(*s*_*i*_|*C*) are centered on the concentrations (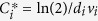) that depend solely on the properties of the compartment and do not depend on the actual concentration of molecules in the sample, many signals contribute little to the result of the assay^16^. In particular, the signals from compartments that contain either a high (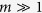) or very small expected number of molecules (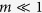), do not contribute much to the result of the assay^13^. This is because in the range of concentrations similar to the actual concentration C of the analyte in the sample, these functions are closely equal to either unity or zero and are almost constant for 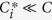 or 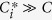, respectively.

## Optimized purely digital assays

We have shown recently that it is possible to minimize the number of compartments needed to estimate the concentration of DNA with a requested precision within a requested range^16^. In essence, we spread the values of C^*^ of each of the compartments in the assay uniformly on the logarithmic scale. This procedure builds an analogue of a positional system, in which the role of digits is played by physical compartments of the examined sample. The positive (1’s) and negative (0’s) signals all have their well-defined positions in writing up the ‘number’ - i.e. the estimate of concentration of the analyte.

## Results

### Combining digital and analogue information

Here we build on the digital assay and supplement it with the information from the real-time (analogue) signals. In our method we use the information on *ct*_*i*_ from the positive compartments to refine the estimate of concentration in the digital scheme. The real-time monitoring of compartments yields the number of cycle (*ct*_*i*_) at which the intensity of fluorescence (which, in this example, is proportional to the number of DNA copies) exceeds a threshold value. Recording of the cycle numbers *ct*_*i*_ and *ct*_*j*_ from two separate compartments, allows us to estimate the ratio of initial number of molecules in them as: *m*_*i*_/*m*_*j*_ = 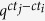. Quite interestingly, our method allows us to determine the amplification factor *q* without any standardized calibration samples using only a known sequence of *d*_*i*_*v*_*i*_ and the “analogue” values *ct*_*i*_ [please see ESI for details].

From the set of (positive) compartments that provided the recording of *ct*_*i*_, we select the one (from now on indexed *ω*) that provided the largest value of the cycle number *ct*_*ω*_, or equivalently, the compartment that contained initially the smallest number of molecules. We know that the reference (*ω*) compartment contained at least a threshold number (*m*_*tr*_) of molecules (because it did yield the positive signal). We also know the set of measurements *ct*_*i*_ from *ω* and from a set of other compartments. We can thus treat *ω* as a reference and calculate that each compartment i contained at least 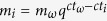 molecules of analyte before amplification. In essence, the set of “analogue” values *ct*_*i*_ allow use to modify (i.e. increase the information content) of the probability density function contributed by each positive compartment.

**Figure 3:**
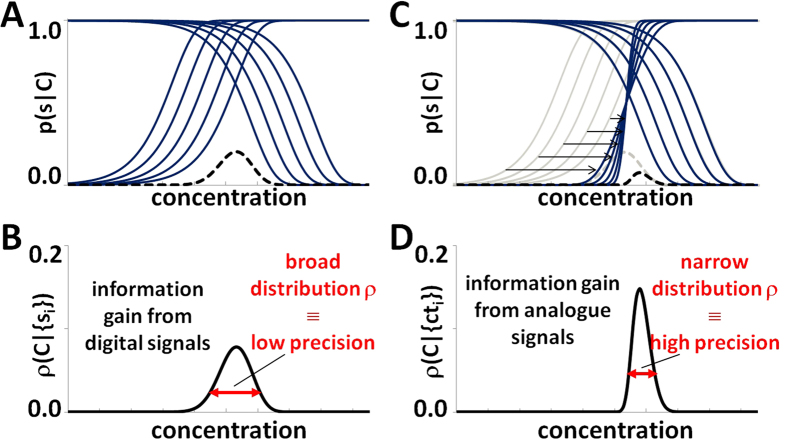
(**A**) The effect of using real-time signal. (**A**) a set (assay) of compartments with geometrical sequence of modulation factor *d*_*i*_*v*_*i*_. Larger compartments yield positive digital signals while the other yield negative digital signals. (**B**) Digital signals from compartments can be used to determine the probability density function of the concentration of the analyte that caused such state of the assay. (**C**) If the digital measurement is accompanied with analogue measurement, sigmoidal functions of probability for positive compartments can be shifted towards the real value of concentration. Hence, they all contribute to the estimation of initial concentration and provide for higher precision. (**D**) The new probability density function of concentration can be calculated, which is narrower than the function based solely on digital measurement, and therefore provides higher precision (lower relative standard deviation) of the estimate of concentration.

In essence, instead of using probabilities that the number of molecules in a compartment is larger than one (standard digital signal), i.e. *m*_*i*_ ≥ 1 we can use 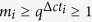, where Δ*ct*_*i*_ = *ct*_*ω*_ − *ct*_*i*_. Therefore, the positive signal can be translated into 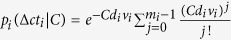. The resulting functions *p*_*i*_(Δ*ct*_*i*_|*C*) qualitatively preserve sigmoidal shape; however, they tend to be steeper and approach (with increasing *m*_*i*_) the shape of the Heaviside step function. Moreover, they are shifted towards the actual concentration C in the sample and therefore increase the information gain, which leads to an improved precision and accuracy of the result (Fig. 3).

### The architecture of the synergistic assay

In order to design the assay (the number and type of partitions of the sample), similarly to the method for rational design of a purely digital assay^16^, we start with the concept of an active stripe, i.e. a set of compartments that contribute information to the estimate of C. As before, we start with the observation, that in a set of non-identical compartments, each of the compartments is centered (i.e. the probability *p(s*|*C*) = 0.5, where the information gain is maximal) at a different value of concentration depending solely on its size and/or dilution. Therefore, only a subset of aforementioned compartments really contribute information to the estimate of the actual concentration; other are too far and therefore are always positive or always negative, bringing no useful information (probability functions shown in Fig. 3A). In other words, very small (or highly diluted) compartments are not useful for probing small concentrations (they are always negative), while big compartments are not useful for probing high concentrations (they are always positive). The subset of compartments that contribute to the information on concentration is called the active stripe. It is necessary to determine its characteristics – the number of compartments it comprises – to design a synergistic assay and test our method.

In order to determine the number of compartments that participate in the assessment of the concentration, we set an arbitrary input concentration *C*_*input*_ and build an assay. We start with a single compartment ‘centred’ on the concentration *C*_*input*_, i.e. 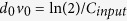. Then, we add symmetrically Δ*N* compartments on each ‘side’, that form geometric sequence: *d*_*i*_*v*_*i*_ = *d*_*0*_*v*_*0*_*x*^*i*^, 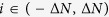. For any sequence (i.e. for any value of the progression factor *x*, the increase of the number 2Δ*N* + 1 of partitions in the active stripe results in an improved assessment of *C*. However, at some value of Δ*N* the precision of the assessment saturates and adding more compartments does not any more effectively improve the information content.

Therefore, the standard deviation *σ* of the estimate of the concentration *E(C*) initially decreases with increasing Δ*N* but at some point saturates at the limit value 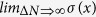 [ESI]. We determined the value of Δ*N* at which *σ(x*) saturates as the optimum number of compartments Δ*N*_*x*_ in the active stripe [ESI]. If we substitute the requested precision *σ*_*max*_ for 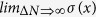 and invert it, we find that the common ratio *x* of the geometric sequence of compartments can be closely approximated as a simple algebraic function of *σ*_*max*_: 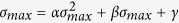 [ESI].

The value of the common ratio *x* provides the sequence *d*_*i*_*v*_*i*_ of the consecutive compartments that provide assessment with a requested precision. The value of Δ*N* provides ‘margins’ of the compartments outside. Knowing that the geometric progression is self similar, it is enough to span the assay to provide the requested precision within the required dynamic range of concentrations 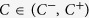 with 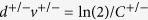, while keeping the values of *x* and Δ*N*.

### Explicit recipe for designing a synergistic PCR assay

To design an assay it suffices to follow the equations below. They use as input the requested dynamic range Ω = C^+^/C^−^ (with *C*^−^ coding the sensitivity limit and *C*^+^ the upper limit of concentration to be assayed) and maximum allowed standard deviation *σ*_*max*_ for the estimates of concentration 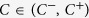:


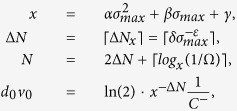


with *α, β, γ, δ* and *ε* being positive constants [ESI].

The above equations (for *x* and Δ*N*) are derived from the close approximations to numerical data, showing the relations between the precision of assessment and input parameters of an assay. They are therefore true for a limited, although practically wide, range of requested precision 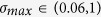. The precision of the assessment can be still improved (i.e. the maximum allowed standard deviation *σ*_*max*_ can be significantly lowered) if the assay is extended with a number of copies of each compartment, forming a set of so-called libraries. The practical teaching on how to prepare such assay is given in the supporting information [ESI].

The equations given above allow a user to design an assay in three simple steps: (i) calculating *x, N* and Δ*N* for requested precision *σ*_*max*_, and dynamic range 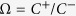, (ii) the volume and dilution of the first (‘largest’) compartment in the sequence *d*_0_*v*_0_ and (iii) determine a geometric sequence comprising *N* partitions of volume and dilution 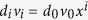 for *i* = 1, …, *N* − 1. The practical guideline how to design a synergistic digital-analogue assay that provides the required dynamic range and precision of the assessment, as well as the instructions on how to analyze the results of the synergistic assays are given in section 1.1 and 1.2 of ESI.

### Verification of the synergistic assay with Monte-Carlo simulations

**Figure 4:**
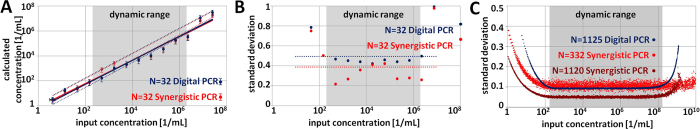
Comparison of the synergistic analogue-digital assay and current state-of-art digital methods. (**A**) Experimental results for 16-compartment multivolume digital (blue points) and synergistic analogue-digital (red points) assays. The value of concentration calculate from the outcome of the assay as a function of a real value of initial concentration is given. Data point shows the averaged results from 12 runs of the assay. The error bars show the standard deviation. The dynamic range of the assays is marked with the gray region. (**B**) The precision of multivolume digital (blue) and synergistic analogue-digital (red) assays given as the relative standard deviation of the estimates within and outside the dynamic range. The dotted lines show the required precision of the assays. (**C**) The comparison of the performance of large digital and synergistic assays. To provide 10% precision of the assessment within 6 log dynamic range, multivolume digital assays (blue) require 1120 compartments, while using synergistic scheme (red) requires only 332 compartments. If we use the same number of compartments, the precision of the assessment is improved.

The synergistic algorithm was compared with state-of-art end-point algorithms (Rational dPCR^16^ and classic dPCR^17^) using experimental and numerical data. First, we compared the performance of the synergistic assay with a rationally designed^16^ purely digital assay. We used a set of N = 16 compartments to build an assay that covers a dynamic range Ω = 10^4^ with a precision *σ* = 70% with end-point readout only (purely digital assay), or the same dynamic range, but improved precision (*σ* = 60%) and accuracy with the synergistic digital-analogue readout (synergistic assays, see Fig. 4A,B). The assays were tested experimentally for 12 different values of concentration within and outside their dynamic range, and each experiment was repeated 12 times. The synergistic assays offer linear relationship between the input concentration and calculated concentration of the analyte (Fig. 4A), which makes them a promising diagnostic tool. The synergistic design was also tested numerically, by means of grand canonical Monte Carlo simulations. We used both synergistic and optimized end-point algorithms to design analytical assays that provide assessment of the concentration with a precision of *σ* = 10%within Ω = 10^6^ dynamic range. The end-point assay required *N* = 1125 compartments while synergistic one required only *N* = 332 compartments, simplifying the laboratory routines. For comparison, a classic digital assay would comprise at least *N* = 400,000 identical compartments to cover the same dynamic range. Moreover, if we use a similar number of compartments as in already optimized end-point assay (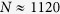), we get 2-fold improvement of precision. The performance of the assays was tested numerically with 10,000 grand canonical Monte Carlo simulations and is presented in Fig. 4C.

### An example of use of a synergistic PCR assay

Although the synergistic assay offers a high performance using a limited number of compartments compared to end-point methods, it still requires more complicated partitioning of the sample than Real-Time assays. The qPCR techniques require only one compartment. They also need a calibration line, which is usually calculated from 15–25 experiments with standardized samples. Still, the number of test volumes for Real-Time assays is usually smaller than for synergistic assays. However, one has to remember that typically the qPCR analysis protocols are optimized for a single set of reagents. This makes the method sensitive to even minor changes in the procedure, for example, differences in the composition of buffers containing the sample and DNA used for calibration. This sensitivity to experimental details renders qPCR prone to errors.

For example, the conventional Real-Time PCR analysis may produce highly scattered results due to just a change of the elution buffer in the nucleic acids isolation protocol. This heavily influences the accuracy of the assay changing the readout of the Real-Time PCR analysis while not signaling the systematic error in the spread of the results (i.e. in reduced precision).

In order to quantify this spread of results, we preformed a conventional qPCR analysis based on high quality IVD certificated clinical Cytomegalovirus detection kit (GeneProof). Positive control DNA from this kit were diluted in three elution buffers from DNA isolation kits (AE elution buffer QIAamp DNA Mini Kit (Qiagen), MBL5 NucleoMag Blood (MACHEREY-NAGEL) and MagJET Whole Blood Genomic DNA Kit (Thermo Scientific) and water to obtain model samples with 25 000 copies of the target DNA per mL. The absolute quantification of sample DNA molecules were calculated using 4 internal kit’s calibrators (from 10 to 10.000 copies per *μ*l) without any dilutions and modifications. The qPCR analysis produced highly scattered results with the following averages: 96 514 [1/mL] for water, 79 368 [1/mL] for the AE buffer, 29 684 [1/mL] for the MBL5 buffer and 24 967 [1/mL] for the MagJET buffer. Thus the change of the buffer in the isolation protocol may heavily influence the accuracy of the assay changing the readout of the qPCR analysis even by a factor of 400% while not signalling the systematic error in the spread of the results (i.e. in reduced precision).

**Figure 5:**
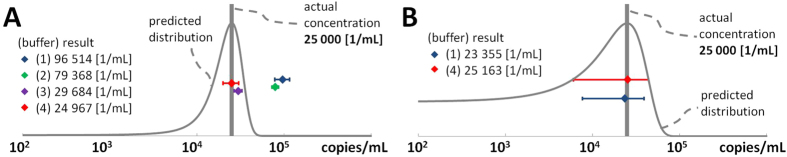
Experimental verification of a 16-compartment digital assay with comparison to the performance of qPCR assays. (**A**) The graph shows the results for the same amount of reference DNA suspended in different elution buffers and quantified with conventional qPCR and with the synergistic PCR algorithm. Tests performed on Applied Biosystems 7500 Fast RT System on the IVD Cytomegalovirus PCR kit (GeneProof) according to the prescription. Elution buffers: (1) water, (2) AE elution buffer QIAamp DNA Mini Kit (Quiagen), (3) MBL5 NucleoMag Blood 200 uL (MACHEREY-NAGEL), (4) MagJET Whole Blood Genomic DNA Kit (Thermo Scientific). The gray line shows the expected distribution of results for Real-Time assay. (**B**) The graph shows the result of 24 runs of the synergistic assay, each on 16 partitions of the amplification mix. We conducted two series of 12 assays on the two elution buffers (1 and 4) that provided the largest difference in the result of the conventional qPCR analysis. The gray line shows the expected distribution of results from the synergistic assay used in the experiment, which should provide 60% precision of assessment. This distribution was also verified using 10,000 Monte-Carlo simulations.

The reason why we observe such a variability in the estimate of concentration provided by qPCR can be the presence of PCR inhibitor in the buffer containing DNA standard. In the experiments we used standardized samples diluted 100 times. If the sample was diluted with water (Fig. 5A, blue point) the inhibiting agent practically disappeared, and therefore higher readout was observed. The agent in question is the ethylenediaminetetraacetic acid (EDTA), present in the buffer, which neutralizes DNases and reduces DNA degradation. The presence of EDTA also lowers the amount of free *Mg*^2+^ ions, which affects the specificity and efficiency of primers. If the sample is diluted 100 times, we eliminate EDTA and make PCR more effective, yielding higher signal. To verify our hypothesis, we repeated the experiment using DNA elution buffers for the dilution of the sample. This time, the results obtained were close to the real value. Hence, only by changing the elution of the clinical sample (either by water or elution buffer), we can accidentally change the estimated provided by qPCR by 400%. Therefore, it is vital to control very accurately the conditions of the reaction, because even a minor alteration significantly changes the final readout. Moreover, a reliable quantitative comparison of the samples purified using different methods may be challenging, or even impossible.

The synergistic PCR algorithm should be insensitive to such problems (Fig. 5B), as it provides absolute quantification and does not require any calibration. To prove it, we performed two series of tests on the samples prepared with the two buffers (water and MagJET buffer) that produced the largest difference in the results from qPCR analysis. In each series we run 12 synergistic analogue-digital tests, using the same qPCR protocol. In a stark contrast to the classic qPCR analysis, the synergistic algorithm correctly estimates the concentration regardless of the method of dilution. The assay was designed to provide the estimates with a 60% standard deviation over a 4 log dynamic range. In each assay we diluted the sample into a sequence of 165 *μ*L partitions and added to each 15 *μ*L of the master mix. The PCR was conducted on the Applied Biosystems 7500 Fast Real-Time System according to the GeneProofs’ prescription. As shown in Fig. 5B, the results of the 24 experiments corroborate very well with the calculated expected distribution of the estimates of concentration. The average of the 12 estimates for the samples diluted with water was 23 355 copies/mL (6,6% off the reference) and the average for the 12 samples diluted with the MagJET buffer was 25 163 copies/mL (0,65% off the reference). The standard deviation of the results corroborates with the predicted precision of the assay span on only 16 independent wells.

Please note that while the 12 repetitions of the 16-well synergistic assay provide a correct average of the estimate of concentration of the target DNA, the precision of such a small (16-well) assay is limited (60%, provided that no inaccuracies from the signal readout or the preparation of the sample are present). If better precision is needed we suggest constructing a bigger assay (i.e. one that uses more wells on the plate) via the algorithms described in this report. For example using 96 wells, one can construct a synergistic assay that delivers an estimate with a precision of 25% over a 4-log dynamic range.

**Figure 6:**
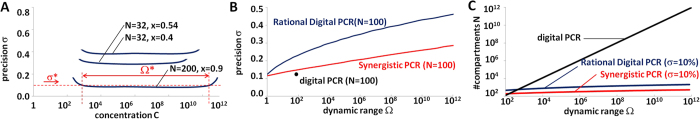
Comparison of the synergistic analogue-digital assay and current state-of-art digital methods: single-volume assay and multi-volume (Rational dPCR) assay. (**A**) The geometrical sequence of volumes/dilutions of compartments described in the synergistic design provides constant information gain in a wide dynamic range, therefore every synergistic assay offers the constant precision of the assessment. (**B**) The performance of the 100-compartment assays. Classic digital assay (black point) offers only one value of precision and dynamic range for a given number of compartments and cannot be tuned. On the other hand, the multi-volume (Rational dPCR; blue line) design allows to ‘trade’ the precision of the assessment for the dynamic range and therefore is more flexible. Synergistic design (red line) offers the same flexibility, but thanks to analogue readout, provides a better precision of the assessment. (**C**) Another advantage of multivolume designs is lower technical requirements in comparison to classic digital assays. In classic digital methods (black line), the number of compartments required for the assessment is directly proportional to the dynamic range, while in digital multivolume (Rational dPCR; blue line) and synergistic (red line) designs this number is proportional to the logarithm of the dynamic range (therefore, it is lowered by orders of magnitude).

Finally, we tested a set of synergistic assays that could be easily implemented in current qPCR devices. The assays were designed to cover the dynamic range of 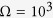 with *σ* = 40% (*N* = 32, *x* = 0.54), and 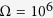 with *σ* = 52% (*N* = 16, *x* = 0.4) and 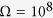 with precision *σ* = 10% (*N* = 200, *x* = 0.9). The performance of the assays is shown in Fig. 6A (each blue line shows a trend drawn from 10,000 grand canonical Monte Carlo simulations for each assay). The observed performance of the assays agrees with analytical predictions, i.e. the precision and dynamic range of each assay is as requested in the design.

The results of the performance tests of synergistic assays shown in Fig. 6 are a good starting point for a more general comparison with current state-of-art solutions: classic dPCR (i.e. end-point measurement using identical compartments) and previously published optimized dPCR (end-point measurement using non-identical compartments^16^). For a fixed number of compartments, digital assays offer assessment within a fixed dynamic range with fixed precision, while multivolume or multidilution designs are more flexible and can be tuned for precision and dynamic range to meet user’s requirements. Moreover, such assays gain a competitive advantage over state-of–art solutions (dPCR and qPCR) thanks both to their high performance taking into account relatively low technical requirements, and low cost.

## Discussion

In conclusion, we have shown that the use of synergistic combination of digital and analogue techniques may bring a new tool in quantitative assaying of DNA load in various samples. The use of information from digital and analogue signals allows to design quantitative assays that provide an absolute (no calibration needed) and precise assessment of concentration of the analyte. Moreover, the technical requirements for running such assays are minimized thanks to the simplified partitioning of the sample (reduction of the number of compartments) and no need for calibration.

The model that we presented also allows to tune independently the dynamic range and precision of the assay and, consequently, construct assays that provide different required precision in different ranges of concentration.

The presented methodology and design of quantitative assays may lead to the development of new, reliable and high-throughput devices for quantitative assaying and find use in the point-of-care applications. From the practical point of view, the reduction of the number of compartments per assay allows running multiple independent assessments using one standard multiwell plates on the standard qPCR machines. For example, one standard 96-well plate is enough to run (i) 3 independent assays that provide dynamic range 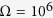 and precision *σ* = 50%, or (ii) 4 assays with 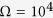 and *σ* = 55%, or (iii) 6 assays with 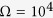 and *σ*=60%. As synergistic PCR assays can readily be designed and executed on standard qPCR devices, they can be used to asses samples for which accuracy of the estimates is important and calibration is difficult or impossible, especially in research applications. In a further perspective, the combination of the new assays with simple detection schemes and high speed amplification systems can be used to introduce an inexpensive system offering precise and absolute quantization taking few minutes from the insertion of the sample, or constructing fast high-throughput systems for the rapid analysis of large number of samples.

## Methods

### Digitalized RT-PCR assay is immune for initial sample buffer composition

#### Materials

All experiment were prepared on IVD certificated PCR kit for Cytomegalovirus detection (GeneProof). Internal calibrator from the kit was used as a DNA template after 400 times diluted in water or 3 different elution buffers from commercially available DNA isolation kits (AE elution buffer from QIAamp DNA Mini Kit (Qiagen), MBL5 elution buffer from NucleoMag Blood (MACHEREY-NAGEL) and MagJET elution buffer from Whole Blood Genomic DNA Kit (Thermo Scientific) to obtain model samples with 25 000 copies of the target DNA per mL.

#### Methods

To compare the traditional qPCR with digital approach three-step amplification protocol was performed in 7500 Fast Real-Time System (Applied Biosystems) according to Cytomegalovirus PCR kit prescription: UGD decontamination 37 degrees for 2 min an initial denaturation at 95 degrees for 10 min. Subsequently, target amplification involved 45 cycles of 5 s at 95 degrees, 40 s at 60 degrees for annealing, then extension for 20 s at 72 degrees. After amplification cycles, PCR products were evaluated for quality using melt curve analysis, which entailed 15 s at 95 degrees, 1 min at 70 degrees, 15 s at 95 degrees and 1 min at 55 degrees.

### Experimental Verification of the Synergistic Analogue-Digital Algorithm

#### Materials

The reaction was performed in a volume of 20 *μL*, consisting of 4.5 *μL* of diluted plasmid DNA, 125 nM of forward and reverse primers (F: tcttgccctctttctgcttc, R: gatcggctcgagaatcattgcg) and 10 *μL* of SensiFAST SYBR No-ROX mix (Bioline).

#### Methods

We used the pJET1.2 plasmid with a fragment of LepA gene cloned from Mycobacterium smegmatis. The initial concentration of DNA was quantified with the use of a NanoDrop device. DNA used for all tests were stored in frozen aliquots.

A three-step amplification protocol was performed in 7500 Fast Real-Time System (Applied Biosystems); an initial denaturation was performed with one cycle at 95 degrees for 10 min. Subsequently, target amplification involved 50 cycles of 15 s at 95 degrees, 25 s at 62 degrees for annealing, then extension for 15 s at 72 degrees. After amplification cycles, PCR products were evaluated for quality using melt curve analysis, which entailed 15 s at 95 degrees, 1 min at 70 degrees, 15 s at 95 degrees and 1 min at 55 degrees.

14 different DNA concentrations were tested from 0.08 to 500,000 DNA molecules in first well (from 0.004 to 25,000 molecules/*μL*). The geometric sequences of the modification factors of compartments comprising tested assays were made via multi-dilution approach, i.e. the volume of all the compartments were same and the dilution factor changed geometrically.

## Additional Information

**How to cite this article**: Debski, P. R. *et al*. Calibration-free assays on standard real-time PCR devices. *Sci. Rep.* **7**, 44854; doi: 10.1038/srep44854 (2017).

**Publisher's note:** Springer Nature remains neutral with regard to jurisdictional claims in published maps and institutional affiliations.

## Supplementary Material

Supporting Information
