# Supplementary material for: Calibration-free assays on standard real-time PCR devices
Source: Sci Rep. 2017 Mar 22;7:44854. doi: 10.1038/srep44854 (PMC5361099; doi:10.1038/srep44854)
Supplement: Supporting Information [file srep44854-s1.pdf]

SUPPORTING INFORMATION FOR:

Calibration-free assays on standard real-time PCR devices.

Pawel R. Debski[1,2], Kamil Gewartowski[2], Seweryn Bajer[2] and Piotr Garstecki[1,2,*]

[1] Institute of Physical Chemistry, Polish Academy of Sciences, Kasprzaka 44/52, 01-224 Warsaw, Poland

[2] Curiosity Diagnostics Sp. z o.o., Kasprzaka 44/52, 01-224 Warsaw, Poland

*Email: garst@ichf.edu.pl

Contents:

1. **Practical guideline how to design a synergistic digital-analogue assay that provides the required dynamic range and precision of the assessment.**
   - 1.1. Detailed description of the construction of an assay with tracking of the identity of each of the compartments.
   - 1.2. Detailed description of the construction of an assay, with a limitation on the minimum dilution factor (or minimum gradation in the volume) between the compartments, with tracking of the identity of each of the compartments.
2. **Step by step instruction on how to analyze the results of a synergistic PCR assay**
3. **Derivation of the model**
   - 3.1. Analytical model of one compartment of the assay – combination of Real-Time and Digital measurement.
   - 3.2. Digital signals.
   - 3.3. Analogue signals - difference in time (or any other) determines the ratios of numbers of molecules.
   - 3.4. Derivation of analytical formulas for assay design
   - 3.5. Sub-ranges.
4. **More improvements**
   - 4.1. Calculation the amplification factor q (if not *a priori* known).
5. **Comparison of synergistic assays with state-of-art methods**
6. **Experimental verification of the algorithms**
   - 6.1. Materials
   - 6.2. Methods
   - 6.3. Results
7. **Digitalized Real-Time PCR assay is immune for initial sample buffer composition**
   - 7.1. Materials
   - 7.2. Methods
   - 7.3. Results

1. Practical guideline how to design a synergistic digital-analogue assay that provides the required dynamic range and precision of the assessment.

The synergistic analytical assays described in this paper can be designed using a set of equations that use as an explicit input the requested precision and the range of concentrations (dynamic range) for which the estimate of concentration of the analyte can be assessed.
The synergistic assays can be run in two ways. The distinction roots from the technical limitations for the generation of compartments that should form a geometric sequence of volumes and dilution factors (or their product). The gradation of the compartment given by the algorithm can be very fine (for example, dilution factor $x = 0.98$ means that the consecutive compartments are diluted by ca. 2%), especially if high precision of the assessment is requested. Such difficulty can be redressed by using assays that comprise sets (libraries) of identical compartments, where the libraries differ by larger dilution factor or ratio of volumes, which is easier to execute.

Below we explain how to design both types of synergistic assays. In section **1.1** we describe optimum synergistic assays, without any technical restrictions on their realization. In section **1.2.** we show how to construct assays comprising libraries of compartments.

1.1. Detailed description of the design of an optimum synergistic assay.

This section details how to design an optimum synergistic assay assuming that trere are no limitations on the gradation in the volume or dilution factor between the compartments. Also, the compartments are tracked, i.e. the volume and dilution factor of each compartment is known.

In order to design a synergistic assay, the user has to determine the requested precision σ_{max} of the assessment (in this case, it is given by the maximum allowed relative standard deviation of the estimate of the initial concentration) and the range of concentrations $C \in (C^-, C^+)$ to be assessed with the requested precision.

Having determined the requirements for the assay, we calculate the gradation in volumes or dilution factor between the compartments, i.e. we calculate the common ratio of the geometric sequence of volumes or dilutions of the compartments comprising the assay. The value of the common ratio is a function of requested precision σ_{max}:

$x = \alpha \cdot \sigma_{max}^2 - \beta \cdot \sigma_{max} + \gamma$,

where $\alpha = 0.5540$, $\beta = 1.6504$ and $\gamma = 1.1135$.

The above expression is valid for the limited range of requested precision $\sigma_{max} \in (0.07,1)$. If the requested precision of the assessment is higher, i.e. $\sigma_{max} < 0.07$, or the gradation x of volume or dilution factor is impossible to implement, the user should additionally determine the finest possible gradation of compartments and follow the instructions in section **1.2** for the design of a synergistic assay comprising libraries of compartments.

Then, we determine the number of compartments belonging to the active stripe:

$\Delta N = \lceil \Delta N_x \rceil = \lceil \delta \cdot \sigma_{max}^{-\varepsilon} \rceil$,

Where $\delta = 2.0532$ and $\varepsilon = 1.3220$,

the total number of compartments in the assay for the requested dynamic range $\Omega = C^+/C^-$:

$N = 2 \cdot \Delta N + \lceil \log_x(1/\Omega) \rceil$,

and the volume and dilution product $d_0 v_o$ of the first compartment in the geometric sequence of compartments that form the assay:

$d_0 v_o = ln2 \cdot x^{-\Delta N} 1/C^-$.

Then, a series of compartments starting from $d_o v_o$ is produced so that the corresponding series of expected numbers of molecules per single partition is a geometric series with quotient x. The said series can be obtained as follows:

1. By changing the volumes v_i of consecutive partitions so that they form a geometric series with quotient x: $v_i = v_o z_i = v_o x^{i-1}$.
2. By changing the dilutions d_i of consecutive partitions so that they form a geometric series with quotient x: $d_i = d_o z_i = d_o x^{i-1}$.
3. More generally, one can use a combination of methods 1) and 2), in this case the products of consecutive dilutions and volumes $d_i v_i$ are adjusted so as to form a geometric series with quotient x: $d_i v_i = d_o v_o z_i = d_o v_o x^{i-1}$.
4. The set of partitions so prepared guarantees determination of concentration in the range $C \in (C^-, C^+)$ with a maximum allowable standard deviation σ_{max}.

1.2. Detailed description of the design of an assay, with a limitation on the minimum dilution factor (or minimum gradation in the volume) between the compartments, with tracking of the identity of each of the compartments.

This section details how to design a synergistic assay with a limitation on the minimum gradation in the volume or dilution factor. The design replaces fine gradation of the volume or dilution between compartments with libraries comprising copies of compartments with the same dilution factor / volume. Also, libraries can be used in order to improve the precision of the assessment.

In order to design a synergistic assay, the user has to determine the range of concentrations $C \in (C^-, C^+)$ to be assessed and, additionally, two aout of three parameters:

a) requested precision σ_{max} of the assessment (in this case, it is given by the maximum allowed relative standard deviation of the estimate of the initial concentration)

b) favourable gradation x (common ratio of the geometric sequence) of volumes and/or dilutions of compartments comprising consecutive libraries, determined by the laboratory equipment, and

a) the number of copies of each compartment N', determined by the laboratory equipment.

Having fixed two out of three parameters, we these two parameters, we first determine the remaining parameter (alternatively, any of the points below).

$$x = \alpha \cdot \sigma_{max}^2 - \beta \cdot \sigma_{max} + \gamma$$

$$\Delta N = \lceil \Delta N_x \rceil = \lceil \delta \cdot \sigma_{max}^{-\varepsilon} \rceil$$

$$N = 2 \cdot \Delta N + \lceil \log_x(1/\Omega) \rceil$$

$$d_0 v_0 = ln2 \cdot x^{-\Delta N} 1/C^-$$

1. having fixed precision σ_{max} and gradation x, we calculate the number of copies N' in the library:

$$N' = \left\lceil \left(\frac{\beta - \sqrt{\beta - 4\alpha(\gamma - x)}}{\alpha \cdot \sigma_{max}} \right)^2 \right\rceil$$

where $\alpha = 0.5540$, $\beta = 1.6504$ and $\gamma = 1.1135$, and the above expression is valid for requested precision $\sigma_{max} < 1$ and common ratio $x \in (0,1)$

2. having fixed the precision σ_{max} and number of copies N', we calculate the gradation x (common ratio of the geometric sequence) of volumes and/or dilutions of compartments comprising consecutive libraries:

$$x = \alpha \cdot \left(\sigma_{max}\sqrt{N'} \right)^2 - \beta \cdot \left(\sigma_{max}\sqrt{N'} \right) + \gamma$$

where $\alpha = 0.5540$, $\beta = 1.6504$ and $\gamma = 1.1135$.

3. having fixed x and N', we calculate the maximum standard deviation σ_{max} of the estimate of the initial concentration:

$$\sigma_{max} = \frac{1}{\sqrt{N'}} \left(\frac{1 - x}{\alpha} \right)^{\frac{1}{\beta}}$$

$$\sigma_{max} = \frac{\beta - \sqrt{\beta - 4\alpha(\gamma - x)}}{\alpha \cdot \sqrt{N'}}$$

where $\alpha = 0.5540$, $\beta = 1.6504$ and $\gamma = 1.1135$, and the above expression is valid for common ratio $x < 1$ and number of copies $N' \geq 1$

Then, we determine the number of compartments belonging to the active stripe:
$\Delta N = \lceil \Delta N_x \rceil = \lceil \delta \cdot \sigma_{max}^{-\varepsilon} \rceil$,
Where $\delta = 2.0532$ and $\varepsilon = 1.3220$,

the total number of compartments in the assay for the requested dynamic range $\Omega = C^+/C^-$:
$N = 2 \cdot \Delta N + \lceil \log_x(1/\Omega) \rceil$,
and the volume and dilution product $d_0 v_o$ of the first compartment in the geometric sequence of compartments that form the assay:
$d_0 v_o = ln2 \cdot x^{-\Delta N} 1/C^-$.

Then, a series of compartments starting from $d_o v_o$ is produced so that the corresponding series of expected numbers of molecules per single partition is a geometric series with quotient x. The said series can be obtained as follows:

1. By changing the volumes v_i of consecutive partitions so that they form a geometric series with quotient x: $v_i = v_o z_i = v_o x^{i-1}$.
2. By changing the dilutions d_i of consecutive partitions so that they form a geometric series with quotient x: $d_i = d_o z_i = d_o x^{i-1}$.
3. More generally, one can use a combination of methods 1) and 2), in this case the products of consecutive dilutions and volumes $d_i v_i$ are adjusted so as to form a geometric series with quotient x: $d_i v_i = d_o v_o z_i = d_o v_o x^{i-1}$.
4. The set of partitions so prepared guarantees determination of concentration in the range $C \in (C^-, C^+)$ with a maximum allowable standard deviation σ_{max}.

2. Step by step instruction on how to analyze the results of a synergistic PCR assay

This section details how to analyze the readout from a synergistic assay. In comparison to classic digital assays, it changes the understanding of the positive signals. In classic digital methods, the positive signal yielded by a compartment or partition $d_i v_i$ states, that in the said partition the number of molecules of the analyte is at least one. This can be interpreted with a probability of obtaining a positive signal, which is a function of initial concentration C:

$$p_i(s_i = 1|C) = 1 - e^{-Cd_i v_i}$$

The synergistic assays use analogue readout to determine the minimum number of the molecules in of analyte m_i present in the said partition , which may be higher than one, i.e. $m_i > 1$. In this case, the probability of obtaining a positive signal, which is a function of initial concentration C, is given by:

$$p_i(s_i = 1|C) = 1 - e^{-Cd_i v_i} \sum_{j=0}^{m_i - 1} [(Cd_i v_i)^j / j!]$$

To analyze the results of the assay, first, the values of $\{m_i\}$ for the positive partitions must be determined.
The analysis starts with collecting the analogue readouts $\{ct_i\}$ for all the positive compartments. Then, the analysis is done as follows:

1. From the set of readouts $\{ct_i\}$ the highest value is chosen and the partition that yield this value will be now called the reference partition ω with readout ct_ω
2. We construct the array of $\{m_i\}$ values as using the following equation:
   $$m_i = q^{\Delta ct_i} = q^{ct_\omega - ct_i}$$
   where q is the amplification factor characteristic for the reaction (it is either *a priori* known, or can be calculated using the instruction give in Section 4.1 of the ESI)
3. Knowing the values $\{m_i\}$ we construct probabilities of obtaining positive signals p_i:
   $$p_i(\Delta ct_i|C) = 1 - e^{-Cd_i v_i} \sum_{j=0}^{m_i - 1} [(Cd_i v_i)^j / j!]$$
4. We also construct probabilities of obtaining negative signals for all negative compartments:
   $$p_i(s_i = 0|C) = e^{-Cd_i v_i}$$
5. Then, we calculate the probability $P(\{s_i\}|C)$ of obtaining the recorded state of the synergistic assay, which is a product of the probability functions for all the compartments:
   $$P(\{s_i\}|C) = \prod_{i=0}^{N-1} p_i(s_i|C)$$
6. Then, we use Bayesian formalism to inverse the product probability to the probability distribution $P(C|\{s_i\})$ of the initial concentration C (under the condition that any *a priori* information about the distribution of C is available):
   $$P(C|\{s_i\}) = P(\{s_i\}|C) / \int_0^\infty P(\{s_i\}|C) dC$$
   for practical reasons (numerical calculation of the integral), the upper limit of integration can be finite, but should be at least one order of magnitude larger than the upper limit of the dynamic range of the assay
7. Knowing the distribution $P(C|\{s_i\})$, one can estimate the initial concentration of the sample as the expected value of this distribution:
   $$E(C) = \int_0^\infty C \cdot P(C|\{s_i\}) dC$$
   again, the upper limit of integration can be finite, but should be at least one order of magnitude larger than the upper limit of the dynamic range of the assay

8. The precision of the estimate can be calculate as the spread (relative standard deviation) of the distribution $\mathrm{P}(\mathrm{C}|\{s_i\})$:

   $$\sigma(C) = \sqrt{E(C^2) - \left(E(C)\right)^2}/E(C)$$

   where

   $$E(C^2) = \int_0^{\infty} \mathrm{C}^2 \cdot \mathrm{P}(C|\{s_i\})dC$$

9. Additionally, it is useful to check the performance of the designed assay numerically. For example, one can run Monte Carlo simulations that take as an input the initial concentration of the analyte (chosen randomly from the dynamic range of the assay), then using random number generators determine the number of molecules of the analyte in every single partition (which has a Poisson distribution with $\lambda = C_A v$), marking positive and negative ones, and then calculate the estimate of the initial concentration using the instructions given in 1-6. As a result, one gets the dependence between the initial concentration and calculated estimated concentration, which is usually 1:1. In some cases, however (especially for small assays), it is slightly tilted. Knowing this dependence from multiple MC simulations, one can use it as a correction function for the estimate of initial concentration from real experiments.

3. Derivation of the model

Below we show the mathematical analysis that leads to the formulation of analytical expressions that can be used as a recipe for the preparation of Synergistic PCR assay. This analysis is a development of our previous works (Debski, P. R., Gewartowski, K., Sulima, M., Kaminski, T. S. & Garstecki, P. Rational design of digital assays. Analytical Chemistry 87, 8203–8209 (2015) and Debski, P. R. & Garstecki, P. Designing and interpretation of digital assays: Concentration of target in the sample and in the source of sample, Biomolecular Detection and Quantification, 10, 24–30 (2016)).

3.1. Analytical model of one compartment of the assay – combination of Real-Time and Digital measurement.

In the discussion below we focus on the application of the method to the qPCR assays. In contrast to the digital scheme, where the only information gained from a given test volume is (the 'digital' yes/no answer) whether there was a finite threshold amount of particles (threshold concentration) reflected by a 'positive' signal, or the opposite case - reflected by a 'negative' signal. This information could be gained by a single measurement from each test volume after a large number of cycles of PCR has been performed (i.e. an end-point signal). In the present invention the method requires iterative measurements of the signal from at least a fraction of the test volumes. According to the method, the number of the cycle (or the time interval from the onset of amplification) is measured, after which the compartment shows fluorescence level higher than a given threshold.

For the analytical model of the assay, it is fair to assume that the molecules of the analyte create a stable and uniform solution. Hence, the expected number of the molecules in one partition of the sample is $E(m) = Cdv$, where C is the concentration of the analyte, v is the volume of this partition and d is the dilution. If the probability of finding a copy of the molecule of interest is not dependant on time or any other parameter, apart from the volume of the partition and concentration, the probability that the number z of molecules of the analyte can is found in this partition is given by the Poisson distribution: $p(m) = e^{-Cdv}(Cdv)^m/m!$. Under this assumption, the positive signal can be translated into a density of probability that a given initial concentration of particles in the sample has caused the positive recording from the partition: $\rho_i(C) = 1 - e^{-Cd_iv_i}\sum_{j=0}^{m_{tr}-1}[(Cd_iv_i)^j/j!]$, while the negative signal can be translated into $\rho_i(C) = e^{-Cd_iv_i}\sum_{j=0}^{m_{tr}-1}[(Cd_iv_i)^j/j!]$. If $m_{tr} = 1$, then the positive signal can be translated into $\rho_i(C) = 1 - e^{-Cd_iv_i}$ and negative into $\rho_i(C) = e^{-Cd_iv_i}$. The important observation is that both probability functions are centered (i.e. have the value of $1/2$) at a concentration determined by the characteristics of the partition only, i.e. in the example, at $C^* = ln(2)/(v_id_i)$.

However, this can only determine the conditional probability of obtaining a microstate for a given concentration. In order to assess the concentration on the basis of a recorded microstate, one has to invert this probability density. In the theory of probability, Bayes' theorem relates inverse representations of the probabilities concerning two events. The input probability is $p(s|C)$, i.e. the output s was observed for a given concentration C. One has to invert this distribution, i.e. to obtain the distribution $\rho(C|s)$ that the concentration C has yielded signal s. If s is a discrete signal, and C is continuous, the distribution $\rho(C|s)$ is given by:

$$\rho(C|s) = \frac{p(s|C) \cdot f(C)}{p(s)}$$

Where $f(C)$ is the distribution function of concentration C. As we do not have any prior knowledge about concentration, $f(C)$ can be described as uniform between arbitrary bounds, i.e. $f(C) = 1/C^{\infty}$, for $C \in (0, C^{\infty})$., and $p(s)$ is the probability of obtaining signal s for any concentration value, which can be also expressed as $p(s) = \int_0^{C^{\infty}} dC \cdot [p(s|C) \cdot f(C)]$. Hence, since $f(C) = const$, $\rho(C|s)$ can be expressed as:

$$\rho(C|s) = \frac{p(s|C) \cdot f(C)}{\int_0^{C^{\infty}} dC \cdot [p(s|C) \cdot f(C)]} = \frac{p(s|C)}{\int_0^{C^{\infty}} dC \cdot [p(s|C)]}$$

3.2. Digital signals.

A classical digital assay allows an independent recording of signals from a set of identical partitions. In essence, supplementing the first observation with an independent recording of a signal from a second test volume improves the information about C . For example, obtaining $s_1 = 1$ and $s_2 = 0$ yields $p_{21}(s_2 = 0, s_1 = 1|C) = p(s_2 = 0|C) \cdot p(s_1 = 1|C)$ and the inverted density of probability $\rho_{21}(C|s_2 = 0, s_1 = 1)$ (Fig. 2c) allows to estimate the value of concentration $E(C)$ and the standard deviation (uncertainty) of this estimation $\sigma(C)$ [ESI]. Generally, information from multiple test-volumes is retrieved from the product of the probability density functions obtained from each of the test-volumes independently: $p(\{s_1, s_2 \dots s_N\}|C) = \prod_{n=1}^{N} p(s_n|C)$. Since in the classic digital assay the compartments are identical, they are also non-distinguishable, reducing the signal to $S = \sum_{n=1}^{N} s_n$. The expected value of concentration $E(C) = f(S)$ and the standard deviation of the estimate $\sigma(C)$ can both be calculated either using various numerical algorithms or analytically.

The result of the whole digital assay (i.e. of the readouts from all the partitons) is given by the product of all the probability densities obtained from individual compartments: $\mathrm{P}(\{s_0, s_1 \dots s_{N-1}\}|C) = \prod_{i=0}^{N-1} \rho(s_i|C)$.

The lowest concentration assessed by a classical digital scheme is $C^- = (Nv)^{-1}$ which corresponds to one molecule of the analyte in the whole assay. The standard deviation of the estimate $E(C)$ changes with S: it is high for extreme values of S and small elsewhere. There is a characteristic point at the concentration $C^+ \sim \frac{5}{v} = \frac{5}{\ln(2)} C^*$ at which $\sigma(S)$ has a deflection point $\frac{d^2\sigma}{dS^2} = 0$ and starts to rise rapidly with further increase of C. Taking this point as a reasonable compromise between a large dynamic range $\Omega = C^+/C^-$ and high precision (i.e. small σ) one obtains: $\Omega(N) = 4.2N$ and $\sigma(N) = 1.45 \cdot N^{-0.479}$ as close fits of the analytical results.

In the classical digital assay, the dynamic range Ω is directly related to N. In other words, obtaining practically large dynamic range, e.g. $\Omega = 10^6$ requires a proportionally large $N \sim \Omega/4 = 250{,}000$. The standard deviation of the estimate from the assay cannot be tuned independently of Ω, i.e. it is not possible to obtain high precision (low σ) in a narrow range of concentrations while using a small N. As a result, in all classic digital assays N needs to be large, at least of the order of 10^4 and preferably of the order of 10^6 or more (*14*,*15*,*16*). This requirement is well reflected in the current technological race towards possibly robust and facile methods for division of the samples into astronomically large number of partitions.

The inefficiency of the classic digital assay follows from the fact that numbering up identical compartments does not allow to gain information about $C \gg C^*$. The classic digital assay is capable of extending the dynamic range to small concentrations, yet in a very inefficient way: with a linear scaling: $C^+/C^- \sim N$ because $C^+ = const$ and $C^- \sim 1/N$. Extension of the range can be done more efficiently by repeating the assay with a diluted sample (*14*) or by preparing a set of classical assays of different volumes of compartments and calculating the estimate on the basis of the set of signals.

We have addressed this problem in the previous work (17), where we have shown that it is possible to simplify the execution of digital analytical methods by running digital tests on a set of non-identical compartments. We described a new class of digital assays that i) maximize the use of information provided by the signal from each of the partitions of the sample, ii) minimize the number of partitions, and iii) allow to independently tune the dynamic range and the precision of the assay. These features are attained via a full use of the digital nature of the analytical scheme: not only in analyzing binary signals, as is done conventionally, but also in employing an analogue of positional coding, as it is done in computing to minimize the number of bits required to code information. In essence, we build positional system, in which the role of digits is played by physical compartments of the examined sample. This provides optimum number of compartments needed to assess the initial concentration with a requested precision within the requested dynamic range.

3.3 Analogue signals - difference in time (or any other) determines the ratios of numbers of molecules.
In this method, in addition to the end-point measurement, an iterative measurement of the signal from each of the test volumes is required. Then, the number of the cycle (or the time interval from the onset of amplification) is measured, after which the compartment shows fluorescence level higher than a given (usually unknown) threshold.
Formally, we assign a real number (t_i) to the interpolated number of cycle at which the signal from the i-th compartment exceeded the threshold value. Measurements of the cycle number for any two positive compartments allows us to calculate the ratio of numbers of molecules in those two compartments, i.e. $m_i/m_j = q^{t_i-t_j}$, where m_i and m_j are the initial numbers of molecules in the two compartments, q is the amplification factor for one cycle (if the amplification factor is not *a priori* known, it can be calculated for the outcome of the assay – see section 3.1). Importantly, this procedure yields only the ratios of the numbers of target particles. It does not yield absolute quantitation. This is why in classic real-time methods, a calibration is needed. In our scheme all we need to derive an estimate of the initial number of particles is an information about the absolute number of particles in at least one of the positive compartments.
In the method we first use the sole digital signals from at least a fraction of the compartments to perform the initial auto-calibration and then use the cycle numbers t_i recorded from the compartments to refine the estimate of the initial concentration of particles in the sample. The refinement of the estimate is basically done by changing the probability function of recorded positive signal using information from RT signal. In essence, numbers of cycles allow to change the hypothesis, that there were $m_i > 1$ molecules in positive compartment.
This information can be used to improve the informational content contributed from positive compartments. From the set of compartments that provided the recording of ct_i, one chooses the one (from now on indexed ω) that provided the largest cycle number ct_ω, or equivalenty, the compartment that contained initially the smallest number of particles.
As we know that the reference (ω) compartment contained at least a threshold number of particles, and we know the set of measurements $\{ct_i\}$, it is known that the compartment j contained at least $m_i = m_\omega q^{ct_\omega - ct_i}$. This shifts the sigmoidal functions $\rho_i(C) = 1 - e^{-Cd_i v_i}$ to be centered closer to the actual concentration in the sample. This procedure allows to improve the precision and accuracy of the result of the assay: $\mathrm{P}(\{s_0, s_1 \ldots s_{N-1}\}|C) = \prod_{i=0}^{N-1} \rho(s_i|C)$.
In essence, instead of using probabilities, that the number of molecules is over a certain threshold, i.e. $m_i \geq 1$ for any positive compartments, we can use $m_i \geq A$, where A is calculated using real-time signal ct. Preferably, we can use probability function for $m_i \geq q^{\Delta\, ct_i}$ (Fig. 3a). However, one has to remember, that the sigmoid function for different m_i differ in shape (Fig. 3a), however, this change can be determined mathematically. Still, we can also use a concept of active stripe from Rational PCR algorithm (17). Also, to provide constant precision for a given dynamic range, the products of volume and dilution $\{d_i v_i\}$ should create a geometrical sequence with common ratio being a function of required precision.

3.4. Derivation of analytical formulas for assay design.

The derivation of analytical formulas for assay design is analogical to previously shown derivation of Rational Design assays (17). It starts with finding an optimal positional system, i.e. an optimal gradation of volumes and/or dilutions of compartments constituting an assay.

The search start from an arbitraty known input concentration C_{input} and centre the assay at a compartment $d_0 v_0 = \ln(2)/C_{input}$ (i.e. set $C_0^* = C_{input}$). Then, on each 'side' a number ΔN of compartments is added (larger and smaller, or less and more diluted). They form a geometric series with the common ratio x. The probability of reading a positive signal depends on the volume of the compartment: $p_i(\Delta\, ct_i|C) = e^{-C d_i v_i} \sum_{j=0}^{m_i-1} \left[(C d_i v_i)^j / j! \right]$ (Fig. 2a in the main text). All the relevant information is provided by a set (an '*active* stripe') of compartments that surround the central on. For a given, fixed value of x, increasing the number $2\Delta N + 1$ of partitions improves the precision of $E(C)$, i.e. the relative standard deviation of the estimate decreases. However, this decrease works only up to a limit. At some point, the compartments are too large or too small (i.e. the information abut C^* is negligible), so the standard deviation σ of $E(C)$ reaches a limit $\lim_{\Delta N \to \infty} \sigma(x)$, which depends solely on x .

This limit $\lim_{\Delta N \to \infty} \sigma(x)$ can be closely approximated by a simple algebraic fit: $\lim_{\Delta N \to \infty} \sigma(x) = ax^2 - bx + c$ with $a = 0.439$, $b = 1.3458$ and *c=0.9960*. As the input for the assay design is the requested maximum standard deviation σ_{max} of the estimate $E(C)$, the term $\lim_{\Delta N \to \infty} \sigma(x)$ should be replaced with σ_{max} and the equation should be inverted. This gives us the formula for the the common ratio x of the geometric sequence of compartments:

$x = \alpha \cdot \sigma_{max}^2 + \beta \cdot \sigma_{max} + \gamma$.

Then, having calculated the common ratio x, one has to determine the number ΔN_x of compartments in the active stripe. It is the lowest integer value of ΔN at which $\sigma(\Delta N)$ saturates, i.e. the derivative of $\sigma(\Delta N)$ with respect to ΔN is zero (for numerical calculations, we set the condition that the derivative should be smaller than than 1/1000). The values of ΔN_x determined in this way can be closely approximated with a simple analytical fit: $\Delta N = \lceil \Delta N_x \rceil = \lceil \delta \cdot \sigma_{max}^{-\varepsilon} \rceil$.

At this point, we have defined the active stripe as a function of the requested precision of the assay.

The assay should also provide the estimate of the concentration $E(C)$ within the requested dynamic range. Since the equations determining the active stripe are true for any value of $d_0 v_0$ (i.e. for any C_{input}), and the geometric progression is self similar, it is enough to span the assay (i.e. add a number of compartments arranged in geometric sequence with common ratio x) within the requested range of concentrations $C \in (C^-, C^+)$, while keeping the required 'margins' of compartments outside $(d^- v^-, d^+ v^+)$, with $d^{+/-} v^{+/-} = \ln(2)/C^{+/-}$

Then, to design a synergistic assay that provides an estimate of concentration within a requested dynamic range with a requested precision, one has to follow a following set equations that use the requested dynamic range $\Omega = C^+/C^-$ and maximum allowed standard deviation σ_{max} of the estimate of C as *explicit* input:

$x = \alpha \cdot \sigma_{max}^2 + \beta \cdot \sigma_{max} + \gamma$

$\Delta N = \lceil \Delta N_x \rceil = \lceil \delta \cdot \sigma_{max}^{-2} + \varepsilon \cdot \sigma_{max}^{-1} - \varphi \rceil$

$N = 2 \cdot \Delta N + \lceil \log_x(1/\Omega) \rceil$

$d_0 v_o = ln2 \cdot x^{-\Delta N} 1/C^-$

with α, β, γ, δ, ε and φ being positive constants:

$\alpha = 0.5540$

$\beta = 1.6504$

$\gamma = 1.1135$

$\delta = 2.0533$

$\varepsilon = 1.3220$

$\varphi = 1.9601$

3.5 Sub-ranges.

For some analytical applications, the required precision of the estimation of concentration may vary for different concentration ranges. Hence, the assay can be tuned in order to provide different precision by changing the common ratio x of the geometric series of volume and dilution product $d_i v_i$. Preferably, the interval (C^-, C^+) can be divided into j subintervals (C_n^-, C_n^+), preferably disjoint ones, in each subinterval the set of values $d_i v_i$ is a decreasing geometric series with quotient x_n, whereas:

For $\sigma_n < \sigma_{n-1}$:

$x_n = \alpha_n \cdot \sigma_{max}^2 + \beta_n \cdot \sigma_{max} + \gamma_n,$

where α_n, β_n and γ_n are constants, and $\alpha_n = 0.5540$, $\beta_n = 1.6504$, $\gamma_n = 1.1135$.

$\Delta N_n = \lceil \delta_n \cdot \sigma_{max}^{-2} + \varepsilon_n \cdot \sigma_{max}^{-1} - \varphi_n \rceil,$

where δ_n, ε_n and φ_n, are constants, and $\delta_n = 0.7343$, $\varepsilon_n = 4.849$ and $\varphi_n = -1.9601$

$N = 2 \cdot \Delta N_n + \lceil \log_x(1/\Omega) \rceil$

$$d_{n_0} v_{n_0} = ln2 \cdot x_n{}^{-\Delta N_n} \left(\frac{1}{C_n^-} \right)$$

For $\sigma_{n_{max}} > \sigma_{n-1_{max}}$

$x_n = \alpha_n \cdot \sigma_{max}^2 + \beta_n \cdot \sigma_{max} + \gamma_n,$

where α_n, β_n and γ_n are constants, and $\alpha_n = 0.5540$, $\beta_n = 1.6504$, $\gamma_n = 1.1135$.

$\Delta N_n = \lceil \delta_n \cdot \sigma_{max}^{-2} + \varepsilon_n \cdot \sigma_{max}^{-1} - \varphi_n \rceil,$

where δ_n, ε_n and φ_n, are constants, and $\delta_n = 0.7343$, $\varepsilon_n = 4.849$ and $\varphi_n = -1.9601$

$N_n = \Delta N_n + \Delta N_{n-1} + \lceil \log_x(1/\Omega) \rceil$

$$d_{n_0} v_{n_0} = ln2 \cdot x_{n-1}{}^{-\Delta N_{n-1}} \left(\frac{1}{C_n^-} \right)$$

Where C^- means the lower limit of the interval for the determination of the unknown concentration C_S, C^+ means the upper limit of the interval for the determination of the unknown concentration C_S, C_n^- means the lower limit of the subinterval with the number n, C_n^+ means the upper limit of the subinterval with the number n, σ_n means the maximum permitted standard deviation of the estimate $E(C_S)$ of the unknown concentration C_S of molecules in the sample in the subinterval $(C_n^- C_n^+)$, n is the subinterval number, running over integers from *1* to j, and i is the partition number, running over integers from *0* to $N - 1$.

4. More improvements

4.1.Calculation the amplification factor q (if not *a priori* known).

The use of real-time signals also allows us to determine the amplification factor (the average of the ratio of the numbers of particles analyte in the test volume after two subsequent cycles or time intervals), if it is not a priori known. The advantage is, that this factor is determined specifically for the current sample and current substrate or apparatus, and each measurement is treated separately.
The calculation of the amplification factor q is done in the following steps:

1. for a known sequence of $\{d_i v_i\}$, the numbers of cycles $\{ct_i\}$, after which threshold signal (level of fluorescence) is observed, are measured
2. observing threshold level o fluorescence is a sign, that the current number of particles in the compartments is equal to some constant value (possibly unknown), hence we can state that $m_{obs} = m_i q^{ct_i}$, where $m_{obs}$is the threshold number and m_i is the initial number of particles
3. the expected value of m_i is equal $d_i v_i C$, where C is constant (it is the real value of concentration) and unknown
4. then, from the measurement of $\{ct_i\}$ and a priori knowledge of $\{d_i v_i\}$, one can plot $ct_i = f(\ln(d_i v_i))$

the gradient of the linear fit to this data is equal to $a = f(q) = 1/\ln(q)$, hence $q = e^{-1/a}$.

5. Comparison of synergistic assays with state-of-art methods

Input parameters		Number of compartments needed for the assessment		
**dynamic range**	**relative precision ***	**Synergistic Assay**	**Rational Digital Assay ****	**Classic Digital Assay *****
4 log	10%	628	794	13000
4 log	15%	267	368	13000
4 log	20%	154	213	13000
4 log	25%	102	139	13000
4 log	30%	73	97	13000
4 log	35%	55	72	13000
4 log	40%	43	55	13000
4 log	45%	34	43	13000
4 log	50%	28	35	13000
6 log	10%	726	1122	1200000
6 log	15%	302	516	1200000
6 log	20%	175	296	1200000
6 log	25%	117	192	1200000
6 log	30%	84	134	1200000
6 log	35%	64	98	1200000
6 log	40%	50	75	1200000
6 log	45%	41	58	1200000
6 log	50%	34	46	1200000
9 log	10%	873	1614	1100000000
9 log	15%	356	737	1100000000
9 log	20%	207	421	1100000000
9 log	25%	139	271	1100000000
9 log	30%	101	189	1100000000
9 log	35%	78	138	1100000000
9 log	40%	62	104	1100000000
9 log	45%	50	81	1100000000
9 log	50%	42	64	1100000000

* the precision of the assessment given as the relative standard deviation of the estimate of initial concentration; the values of precision given here do not include any dilution or signal readout errors (in practical use, when the inaccuracies of the experimental system are taken into account, the precision may be worse)

** as described in Debski, P. R., Gewartowski, K., Sulima, M., Kaminski, T. S. & Garstecki, P. *Rational design of digital assays*. Analytical Chemistry 87, 8203–8209 (**2015**)

*** as described in Debski, P. R. & Garstecki, P. *Designing and interpretation of digital assays: Concentration of target in the sample and in the source of sample*. Biomolecular Detection and Quantification 10, 24-30 (**2016**)

Table T1. The comparison of the technical requirements, i.e. the number of compartments needed for the assessment oft he initial concentration of the analyte with requested parameters (dynamic range and relative precision of the estimate), for running synergistic assays and state of-art-schemes: Rational Digital assay and classic digital (single-volume) assay. The synergistic scheme offers an almost 2-fold reduction of the number of compartments required for the assessment compared to Rational Digital scheme. It also requires by orders of magnitude less compartments compared to single-volume digital scheme. What is more, in the digital assays designed in single-volume scheme, the precision of the assessment and the dynamic range is fixed.

6. Experimental verification of the model

6.1 Materials

The reaction was performed in a volume of 20 µL, consisting of 4.5 µL of diluted plasmid DNA, 125 nM of forward and reverse primers (F: tcttgccctctttctgcttc, R: gatcggctcgagaatcattgcg) and 10 µL of SensiFAST SYBR No-ROX mix (Bioline).

6.2 Methods

We used the pJET1.2 plasmid with fragment of LepA gene cloned from *Mycobacterium smegmatis*. The initial concentration of DNA was quantified with the use of a NanoDrop device. DNA used for all tests were stored in frozen aliquots.

A three-step amplification protocol was performed in 7500 Fast Real-Time System (Applied Biosystems); an initial denaturation was performed with one cycle at 95°C for 10 min. Subsequently, target amplification involved 50 cycles of 15 s at 95°C, 25 s at 62°C for annealing, then extension for 15 s at 72°C. After amplification cycles, PCR products were evaluated for quality using melt curve analysis, which entailed 15 s at 95°C, 1 min at 70°C, 15 s at 95°C and 1 min at 55°C.

14 different DNA concentrations were tested from 0.08 to 500,000 DNA particles in first well (from 0.004 to 25,000 particles/µl).

The geometric sequences of the modification factors of compartments comprising tested assays were made via multi-dilution approach, i.e. the volume of all the compartments was same and the dilution factor changed geometrically.

6.3 Results

**Input C**	**25 000 000 [1/mL]**		**2 250 000 [1/mL]**		**675 000 [1/mL]**		**202 500 [1/mL]**	
**#run**	calculated C [1/mL]	relative precision	calculated C [1/mL]	relative precision	calculated C [1/mL]	relative precision	calculated C [1/mL]	relative precision
**1**	all positive	N/A	7 750 000	< 100%	540 000	89%	83 000	43%
**2**	all positive	N/A	7 740 000	< 100%	540 0000	86%	92 000	59%
**3**	all positive	N/A	550 000	85%	160 000	35%	276 000	63%
**4**	all positive	N/A	290 000	57%	180 000	65%	89 000	63%
**5**	all positive	N/A	7 750 000	< 100%	190 000	34%	33 000	61%
**6**	all positive	N/A	8 880 000	< 100%	400 000	73%	175 000	68%
**7**	all positive	N/A	7 750 000	< 100%	400 000	73%	125 000	58%
**8**	all positive	N/A	7 760 000	< 100%	190 000	37%	280 000	14%
**9**	all positive	N/A	7 750 000	< 100%	180 000	68%	188 000	61%
**10**	all positive	N/A	7 780 000	< 100%	410 000	70%	400 000	71%
**11**	all positive	N/A	7 750 000	< 100%	760 000	11%	143 000	53%
**12**	all positive	N/A	7 750 000	< 100%	200 000	24%	227 000	39%
**average**	**-**		**7 030 000**		**320 000**		**168 000**	
**relative spread**	**-**		**32%**		**47%**		**44%**	

**input C**	**60750 [1/mL]**		**18225 [1/mL]**		**5460 [1/mL]**		**1640 [1/mL]**	
**#run**	calculated C [1/mL]	relative precision	calculated C [1/mL]	relative precision	calculated C [1/mL]	relative precision	calculated C [1/mL]	relative precision
**1**	80 800	8%	8 800	39%	1 620	62%	1 240	51%
**2**	65 200	61%	8 500	56%	2 280	40%	1 690	50%
**3**	22 400	64%	11 200	66%	3 830	47%	4 080	44%
**4**	12 100	55%	3 400	55%	790	68%	11 100	68%
**5**	8 700	61%	8 700	60%	3 560	53%	1 310	54%
**6**	45 900	60%	5 700	66%	6 260	55%	1 540	67%
**7**	42 700	67%	5 800	65%	3000	66%	620	62%

**8**	24 600	35%	11 200	66%	15 520	60%	1 170	53%
**9**	25 800	53%	10 000	49%	11 550	55%	1 160	41%
**10**	84 400	68%	16 900	61%	4 510	62%	620	62%
**11**	43 000	66%	8 200	58%			1 270	50%
**12**	25 800	53%					1 550	37%
**average**	**38 900**		**8 700**		**4 580**		**1 560**	
**relative spread**	**55%**		**23%**		**69%**		**60%**	

**input C**	**492 [1/mL]**		**148 [1/mL]**		**44 [1/mL]**		**13 [1/mL]**	
**#run**	calculated C [1/mL]	relative precision	calculated C [1/mL]	relative precision	calculated C [1/mL]	relative precision	calculated C [1/mL]	relative precision
**1**	10 000	8%	370	23%	400	68%	20	98%
**2**	1 490	25%	150	48%	110	69%	34	71%
**3**	820	64%	3 960	5%	83	64%	20	98%
**4**	1 640	61%	80	64%	51	76%	39	71%
**5**	490	53%	660	25%	20	95%	20	98%
**6**	1 090	46%	110	69%	51	76%	6.6	> 100%
**7**	2 050	21%	730	19%	51	76%	20	98%
**8**	3 460	11%	210	66%	40	71%	34	71%
**9**	510	52%	250	54%	39	71%	20	98%
**10**	540	49%	50	75%	83	64%	34	71%
**11**	1 000	44%	80	64%	51	76%	6.6	> 100%
**12**	2 960	68%	130	57%	34	71%	6.6	> 100%
**average**	**1 560**		**280**		**60**		**21**	
**relative spread**	**64%**		**85%**		**40%**		**48%**	

**input C**	**4 [1/mL]**		**1 [1/mL]**	
**#run**	calculated C [1/mL]	relative precision	calculated C [1/mL]	relative precision
**1**	5.3	> 100%	all negative	N/A
**2**	19	100%	all negative	N/A
**3**	36	71%	all negative	N/A
**4**	28	> 100%	all negative	N/A
**5**	5.3	> 100%	all negative	N/A
**6**	5.3	> 100%	all negative	N/A
**7**	5.3	> 100%	all negative	N/A
**8**	5.3	> 100%	all negative	N/A
**9**	5.3	> 100%	all negative	N/A
**10**	19	> 100%	all negative	N/A
**11**	19	> 100%	all negative	N/A
**12**	28	> 100%	all negative	N/A
**average**	**13.9**			**-**
**relative spread**	**69%**			**-**

Table T2. Experimental results of the verification oft he performance of Synergistic PCR. The readouts from the Synergistic PCR analysis of the sample with the concentration of target DNA changing from 1 [1/mL] to 25 000 000 [1/mL], which coverst he dynamic range of an assay, are given. For each concentration, the experiment was repeated 12 times. The averages of the readouts and relative spreads of the results are given extereme values (lowest and highest) are neglected.

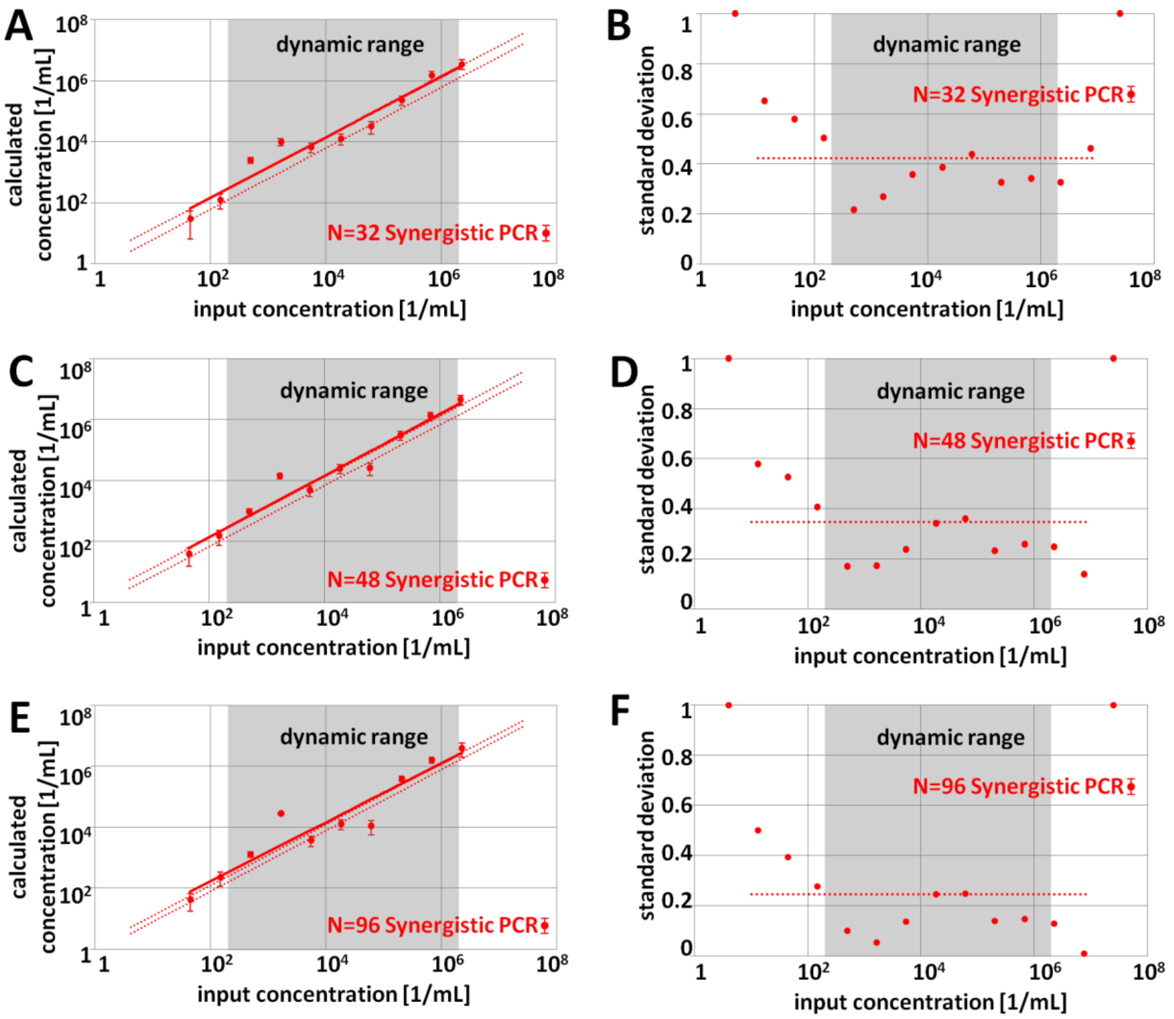

Figure S1. Experimental verification of the synergistic algorithm.(a,c,e) The estimated concentration of DNA as a function of known input concentration used in the experiments. Each data point is an average over the number of independent runs of the same assay design for each concentration value (e.g. for 32-partition assays, the data point is an average over 6 results, for 48-partitions assays an average over 4 results, and an average over 2 results for the 96-partition assays). The assays presented here were constructed using the copies of a “basic” 16-partition (and 16 dilutions) assay: 32-partition assay comprises of two copies of the basic assay, i.e. there are two copies of each of the 16 dilutions, 48-partition assays comprises of four copies, and the 96-partition assay comprises of 8 copies. Therefore, the synergistic scheme allows for the improvement of the precision of the assessment using simple procedures, such as adding copies of each compartment in the assay. Error bars indicate the standard deviation of the estimate, calculated over the set of results, drawn as one standard deviation away from the point (i.e. corresponding to the 68% confidence interval). The solid red line is a power fit to the averaged results. The dotted red lines indicate the region of expected outputs of the digital assay, given by nominal input concentration plus/minus one standard deviation expected from the assay. The grey-shaded region marks the designed dynamic range of the assay. (b, d, f) Standard deviation of the estimates from our algorithms as a function of the input concentration, while the red dotted line marks the expected standard deviation for a given digital assay, within the dynamic range (again, marked with the grey-shade)

7. Digitalized Real-Time PCR assay is immune for initial sample buffer composition

7.1 Materials

All experiment were prepared on IVD certyficated PCR kit for Cytomegalovirus detection (GeneProof). Internal calibrator form the kit was used as a DNA template after 400 times diluted in water or 3 different elution buffers from commercially available DNA isolation kits (AE elution buffer from QIAamp DNA Mini Kit (Qiagen), MBL5 elution buffer from NucleoMag Blood (MACHEREY-NAGEL) and MagJET elution buffer from Whole Blood Genomic DNA Kit (Thermo Scientific) to obtain model samples with 25 000 copies of the target DNA per mL.

7.2 Methods

To compare the traditional Real-Time PCR with digital approach three-step amplification protocol was performed in 7500 Fast Real-Time System (Applied Biosystems) according to Cytomegalovirus PCR kit prescription: UGD decontamination 37°C for 2 min an initial denaturation at 95°C for 10 min. Subsequently, target amplification involved 45 cycles of 5 s at 95°C, 40 s at 60°C for annealing, then extension for 20 s at 72°C. After amplification cycles, PCR products were evaluated for quality using melt curve analysis, which entailed 15s at 95°C, 1 min at 70°C, 15 s at 95°C and 1 min at 55°C.

7.3 Results

The tables below contain all the redaouts from the real-time PCR.

#trial	Water (1st series)	Water (2nd series)	MagJet (1st series)	MagJet (2nd series)	AE	MBL5
1	115 000	118 000	21 000	29 000	90 000	25 000
2	112 000	79 000	27 000	30 000	81 000	30 000
3	109 000	71 000	25 000	25 000	84 000	36 000
4	131 000	106 000	22 000	24 000	86 000	28 000
5	114 000	88 000	29 000	25 000	72 000	27 000
6	95 000	70 000	26 000	27 000	77 000	30 000
7	121 000	84 000	26 000	25 000	70 000	31 000
8	123 000	95 000	24 000	24 000	74 000	31 000
9	121 000	75 000	29 000	28 000		
10	104 000	75 000	23 000	23 000		
11	108 000	75 000	21 000	32 000		
12	117 000	85 000	26 000	45 000		
13	116 000	85 000	23 000	32 000		
14	114 000	77 000	21 000	35 000		
15	113 000	77 000	20 000	23 000		
16	112 000	69 000	21 000	25 000		
17	118 000	77 000	22 000	22 000		
18	83 000	89 000	20 000	21 000		
19	85 000	74 000	19 000	21 000		
20	93 000	88 000	19 000	19 000		
average value	97 000 [1/mL]		25 000 [1/mL]		79 000 [1/mL]	30 000 [1/mL]
relative spread	19%		20%		9%	10%

Table T3. Experimental results of the verification of Real-Time PCR. The readouts from the real-time PCR analysis of the sample containing the same initial number of target DNA sequence are given. The only change in the reaction procedure was the elution buffer used: water, MagJet, AE and MBL5. The averages of the readouts and the relative spreads of the results are given.

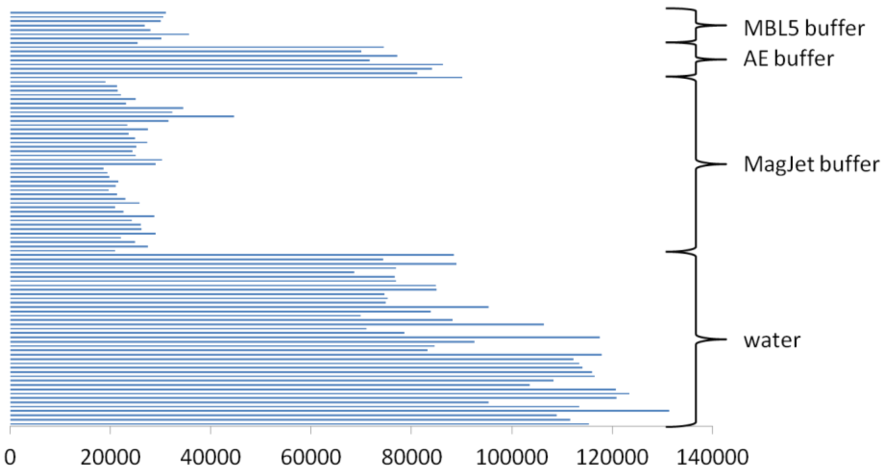

Figure S2. Experimental results of the verification of Real-Time PCR. The readouts from the real-time PCR analysis of the sample containing the same initial number of target DNA sequence are given.

		**Water**		**MagJet**	
**#run**	input C [1/mL]	calculated C [1/mL]	relative precision	calculated C [1/mL]	relative precision
**1**	25000	54 000	73%	3 700	70%
**2**	25000	22 000	74%	3 800	54%
**3**	25000	38 000	70%	57 000	63%
**4**	25000	24 000	33%	57 000	70%
**5**	25000	24 000	67%	18 000	43%
**6**	25000	45 000	39%	16 000	46%
**7**	25000	21 000	75%	15 000	46%
**8**	25000	23 000	70%	26 000	63%
**9**	25000	6 000	36%	6 000	63%
**10**	25000	950	50%	48 000	82%
**11**	25000	12 000	57%	27 000	32%
**12**	25000	10 000	65%	24 000	67%
**Average**		**23 400 [1/mL]**		**25 200 [1/mL]**	

Table T4. Experimental results of the verification of the Synergistic PCR scheme. The readouts from the Synergistic PCR analysis of the sample containing the same initial number of target DNA sequence are given. The only change in the reaction procedure was the elution buffer used: water and MagJet. The averages of the readouts and the relative spreads of the results are given.

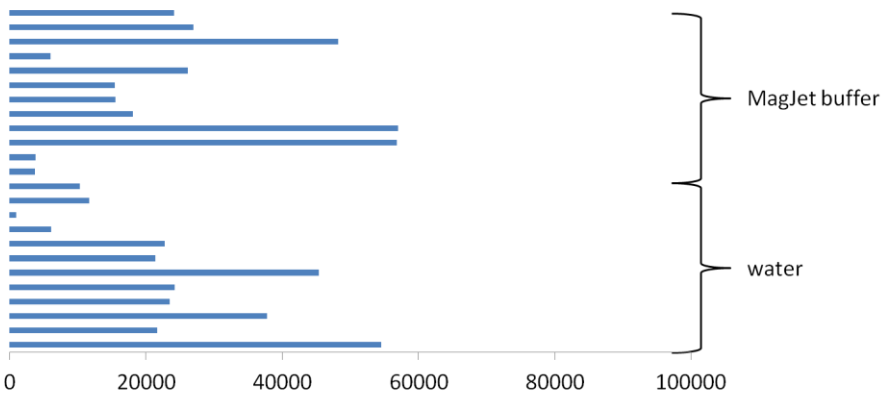

Figure S3. results of the verification of the Synergistic PCR scheme. The readouts from the Synergistic PCR analysis of the sample containing the same initial number of target DNA sequence are given.
